# Characteristics and interplay of esophageal microbiota in esophageal squamous cell carcinoma

**DOI:** 10.1186/s12885-022-09771-2

**Published:** 2022-06-24

**Authors:** Zheng Lin, Wenqing Rao, Zhisheng Xiang, Qiaoyan Zeng, Shuang Liu, Kaili Yu, Jinsong Zhou, Jianwen Wang, Weilin Chen, Yuanmei Chen, Xiane Peng, Zhijian Hu

**Affiliations:** 1 Department of Epidemiology and Health Statistics, Fujian Provincial Key Laboratory of Environment Factors and Cancer, School of Public Health, Fujian Medical University, Fuzhou, 350122 China; 2 Department of Epidemiology, Fujian Medical University Cancer Hospital, Fujian Cancer Hospital, Fuzhou, 350014 China; 3 Department of Digestive Endoscopy, Anxi County Hospital, Anxi, 362400 China; 4 Department of Radiation Oncology, Zhangzhou Affiliated Hospital of Fujian Medical University, Zhangzhou, 363000 China; 5 Department of Thoracic Surgery, Fujian Medical University Cancer Hospital, Fujian Cancer Hospital, Fuzhou, 350014 China; 6 Key Laboratory of Ministry of Education for Gastrointestinal Cancer, Fujian Medical University, Fuzhou, 350122 China

**Keywords:** Esophageal squamous cell carcinoma, Microbiota, Co-occurrence network, PICRUSt2

## Abstract

**Background:**

Esophageal microbiota may influence esophageal squamous cell carcinoma (ESCC) pathobiology. Therefore, we investigated the characteristics and interplay of the esophageal microbiota in ESCC.

**Methods:**

We performed 16S ribosomal RNA sequencing on paired esophageal tumor and tumor-adjacent samples obtained from 120 primarily ESCC patients. Analyses were performed using quantitative insights into microbial 2 (QIIME2) and phylogenetic investigation of communities by reconstruction of unobserved states 2 (PICRUSt2). Species found to be associated with ESCC were validated using quantitative PCR.

**Results:**

The microbial diversity and composition of ESCC tumor tissues significantly differed from tumor-adjacent tissues; this variation between subjects beta diversity is mainly explained by regions and sampling seasons. A total of 56 taxa were detected with differential abundance between the two groups, such as *R. mucilaginosa*, *P. endodontalis*, *N. subflava*, *H. Pylori*, *A. Parahaemolyticus*, and *A. Rhizosphaerae*. Quantitative PCR confirmed the enrichment of the species *P. endodontalis* and the reduction of *H. Pylori* in tumor-adjacent tissues. Compared with tumor tissue, a denser and more complex association network was formed in tumor-adjacent tissue. The above differential taxa, such as *H. Pylori*, an unclassified species in the genera *Sphingomonas*, *Haemophilus*, *Phyllobacterium*, and *Campylobacter*, also participated in both co-occurrence networks but played quite different roles. Most of the differentially abundant taxa in tumor-adjacent tissues were negatively associated with the epidermal growth factor receptor (EGFR), erb-b2 receptor tyrosine kinase 2 (ERBB2), erb-b2 receptor tyrosine kinase 4 (ERBB4), and fibroblast growth factor receptor 1 (FGFR1) signaling pathways, and positively associated with the MET proto-oncogene, receptor tyrosine kinase (MET) and phosphatase and tensin homolog (PTEN) signaling pathways in tumors.

**Conclusion:**

Alterations in the microbial co-occurrence network and functional pathways in ESCC tissues may be involved in carcinogenesis and the maintenance of the local microenvironment for ESCC.

**Supplementary Information:**

The online version contains supplementary material available at 10.1186/s12885-022-09771-2.

## Introduction

Esophageal cancer is a malignant cancer and contributes to disease burden, especially in developing countries. In 2020, there were 604,100 new cases of esophageal cancer globally [1]: China had the highest number of incident cases, accounting for up to 53% of new cases [2]. There are two main histological subtypes of esophageal cancer, esophageal squamous cell carcinoma (ESCC) and esophageal adenocarcinoma (EAC), among which ESCC accounts for more than 85% of global esophageal cancer cases [3]. ESCC develops from the squamous epithelial cells that make up the inner lining of the esophagus. The etiology of ESCC is multifactorial and still not well elucidated, which restricts the effective prevention of this disease. Thus, there is an urgent need to clarify the pathogenesis of ESCC and to explore new diagnostic and therapeutic possibilities.

The microbiome is an emerging area in the etiological exploration of various diseases. A balance of the microbiome is strongly associated with a variety of human diseases: the gastrointestinal microbiota plays an indispensable role in maintaining the health of humans [4]. The normal microenvironment is conducive to maintaining the steady state of the esophagus, and dysbiosis of the microbiota can lead to the occurrence and development of ESCC [5, 6]. However, there are insufficient studies on the changes in esophageal microbiota in ESCC, and the findings of these studies are also inconsistent [7, 8]. The earliest findings are that lower microbial richness in the upper digestive tract is independently associated with esophageal diseases [9]. However, these upper digestive microbiota may not colonize the esophagus. Then, Yang investigated the differences in esophageal microbiota in ESCC (*n* = 18) and patients with physiologically normal esophagus (*n* = 11) by 16S rRNA profiling [8]. In our previous studies, we detected the esophageal microbiota associated with alcohol consumption (a well-known risk factor for ESCC) [10] and prognosis [11] in ESCC. Shao characterized the microbial communities of paired tumor and nontumor samples from only 67 patients with ESCC in northern China [7]. Though the above studies explored the esophageal microbiota, the sample sizes were too small to find differences in diversity with adequate power [12] and did not take other factors that influenced the microbiota into consideration, including different regions and sampling seasons.

Recently, factors, including the region [13], sampling season [14], and interactions between microbes [15], have been shown to influence microbiota composition. He et al. determined that host location showed the strongest associations with microbiota variations [13]. Moreover, there are significant shifts in human microbiome composition across different seasons [14]. In addition, microbes involved in a variety of behaviors involving complex cooperation and communication may be involved in the development of human diseases, and no studies have reported the interaction network of the esophageal microbiota thus far. Hence, it is essential to consider these factors in microbiome studies.

Therefore, to identify the impact of different regions and sampling seasons on the esophageal mucosal microbiota of ESCC, we conducted a study and performed high-throughput profiling of the esophageal mucosal microbiota in paired tumor and tumor-adjacent tissues in the same ESCC cases. Additionally, we detected the microbial interactions by co-occurrence networks and their functional effects on the host.

## Methods

### Study population

We performed a hospital-based retrospective study of 120 patients pathologically diagnosed with primary ESCC between February 2013 and October 2017 at Fujian Provincial Cancer Hospital and Zhangzhou Municipal Hospital (Supplementary file 1). Subjects were chosen according to the following criteria. Inclusion criteria: (a) underwent esophagectomy surgery; (b) pathologically diagnosed with primary ESCC; (c) tumor stage clarified with number of harvested lymph nodes (HLN) ≥ 20; (d) undergoing neither preoperative radiotherapy nor chemotherapy; (e) no antibiotic use through preoperative two months; (g) no record of other infectious diseases; and (h) resident of Fujian Province for more than 10 years. Exclusion criteria: (a) incomplete clinicopathological data and nonavailability of tissue samples; (b) metastatic malignancy or recurrent esophageal cancer; (c) received pharmacotherapy (such as oral, intramuscular, and intravenous antibacterial drugs, various probiotics or other drugs affecting the microbiota) within a month. Written informed consent was obtained from all the patients. The study was approved by the Ethics Committee of Fujian Medical University (approval no. 201495).

### Demographic and clinical information

Basic information for all the participants was collected through a detailed questionnaire that included sociodemographic status, dietary habits, daily physical activity, smoking status, alcohol consumption, family history of cancer and gastrointestinal symptoms. Clinicopathological features including tumor location, and tumor, node, and metastasis (TNM) stage for each patient were also collected from their respective medical records.

### Sample collection and preservation

Paired tumor and tumor-adjacent tissue samples were obtained from each patient immediately after surgical resection in the operating room. The tumor-adjacent tissue samples were from an area at a distance of 3 cm from the tumor tissue. The tissue samples were cut into small pieces, placed in autoclaved cryovials, stored in liquid nitrogen for 1 h, and, then transferred to a − 80 °C refrigerator for storage. All samples were evaluated by pathological hematoxylin-eosin (HE) staining.

### Bacterial DNA extraction and 16S rRNA sequencing

The sodium dodecyl sulfate (SDS) method was used to extract bacterial DNA from the samples. The extracted DNA was quantitatively detected by a Qubit fluorometer (Invitrogen, America), and the results were acceptable. Each extraction was performed with a blank buffer control to detect contaminants from either reagents or other unintentional sources. However, the negative controls detected too little DNA to prepare the library and hence were not sequenced.

Amplification of the 16S ribosomal RNA (rRNA) gene used primers targeting regions V3–V4, which included a forward primer (341F: 5′-CCTAYGGGRBGCASCAG-3′) and reverse primer (806R: 5′-GGACTACNNGGGTATCTAAT-3′). The sequencing platform was the HiSeq2500 PE250 (Illumina, America).

### Quantitative PCR (qPCR) validation

DNA from samples was diluted 10- and 100-fold, respectively, in PCR grade water to facilitate downstream analysis. Relative abundance was calculated by the ΔCt method. The cycle threshold (Ct) values for *P. endodontalis* and *H. pylori* were normalized using a primer set for the total bacteria. The primer sequences for each assay were as follows: *P. endodontalis* forward primer, 5′-CGGTAATACGGAGGATACG-3′; *P. endodontalis* reverse primer, 5′-TTCAACGGCAAGACTACA-3′; *H. pylori* forward primer, 5′-GGTGAGTAACGCATAGGT-3′; *H. pylori* reverse primer, 5′-CTGATAGGACATAGGCTGAT-3′; Eubacteria 16S forward primer, 5′-GGTGAATACGTTCCCGG-3′; Eubacteria 16S reverse primer, 5′- TACGGCTACCTTGTTACGACTT-3′. The fold difference (2^-ΔΔCt^) in *P. endodontalis* and *H. pylori* abundance in tumor versus tumor-adjacent tissue was calculated by subtracting ΔCt_tumor_ from ΔCt_tumor-adjacent_, where ΔCt is the difference in threshold cycle number for the test and reference assay. Amplification was performed with the ABI QuantStudio™ 5 RealTime PCR system (Applied Biosystems) using the following reaction conditions: 30 sec at 95 °C, and 40 cycles of 5 sec at 95 °C and 30 sec at 60 °C.

Total RNA was extracted using TRIzol total RNA isolation reagent (ACCURATE BIOTECHNOLOGY, HUNAN,Co.,Ltd). RNA was used to reverse transcribe cDNA using an Evo M-MLV RT Kit with gDNA Clean for qPCR (ACCURATE BIOTECHNOLOGY, HUNAN,Co.,Ltd). Phosphatase and tensin homolog (PTEN) (forward primer, 5′- GAGGGCCAGGTCATAAATAA-3′, reverse primer, 5′- ACCATAAAATGTAAGCAAGGC-3′) and glyceraldehyde-3-phosphate dehydrogenase (GAPDH) (forward primer, 5′-GCACCGTCAAGGCTGAGAAC-3′, reverse primer, 5′-TGGTGAAGACGCCAGTGGA-3′) mRNA levels were determined using quantitative reverse transcription PCR (qRT-PCR).

### Sequence data processing

Raw sequencing data from patients with ESCC were imported into quantitative insights into microbial ecology (QIIME2–2020.02) [16] and processed using the DEBLUR algorithm to denoise and then to infer exact amplicon sequence variants (ASVs). The detailed analysis workflow is presented in Supplementary file 1. To provide better resolution and limit the false discovery rate (FDR) penalty on statistical tests, the low abundance features (with total ASV counts less than 100 or detected in less than 20 samples) were filtered before the differential abundance analysis (955 features left) (Table S1).

### Statistical analysis

Questionnaires and clinicopathological data were double-entered into EpiData (version 3.1, Denmark). The demographic and baseline clinical features were displayed using n (%). The individuals’ risk index of ESCC was calculated by variables including age, smoking, drinking, eating speed, hot food, pickled food, and fruit from the questionnaire (Supplementary file 2). All statistical analyses were evaluated using R software (R version 4.0.2), and a two-tailed *P* < 0.050 was considered statistically significant.

The detailed analyses of microbial diversity are shown in Supplementary file 1. The analysis of composition of microbiomes 2 (ANCOM2) tests [17] were performed to detect the differential abundance in different tissue groups. For the functional prediction, the phylogenetic investigation of communities by reconstruction of unobserved states 2 (PICRUSt2) [18] pipeline was used to generate predictions for EC numbers and MetaCyc pathways. The strength of the edges of the microbial co-occurrence network was assessed by the SparCC algorithm [19], and the interaction network diagram was visualized with Cytoscape [20]. The top hub taxa were assessed by the plugin cytoHubba [21] in Cytoscape. Then, we selected four well-established pathogenesis enzyme genes in ESCC according to Lin [22] from the Kyoto Encyclopedia of Genes and Genomes (KEGG) database [23]. Then, the Spearman correlation was performed to explore the association between the differential taxa and four enzyme genes. The DESeq algorithm [24] was applied to calculate the differential MetaCyc pathways and visualized by volcano plot. Then, the Spearman correlation between differential taxa and differential MetaCyc pathways was calculated.

## Results

### Basic characteristics of all participants

The demographic and clinical data of the study population are reported in Table S2. A total of 120 ESCC patients were recruited from two independent clinical centers. There were 89 males and 31 females. The median age was 61 years. The majority of the tumors originated from the middle (*n* = 57) and lower (*n* = 52) esophagus. Most of the patients had advanced tumors (*n* = 72). Approximately 85 cases had a high risk index.

### Esophageal microbial diversity analysis

**Fig. 1:**
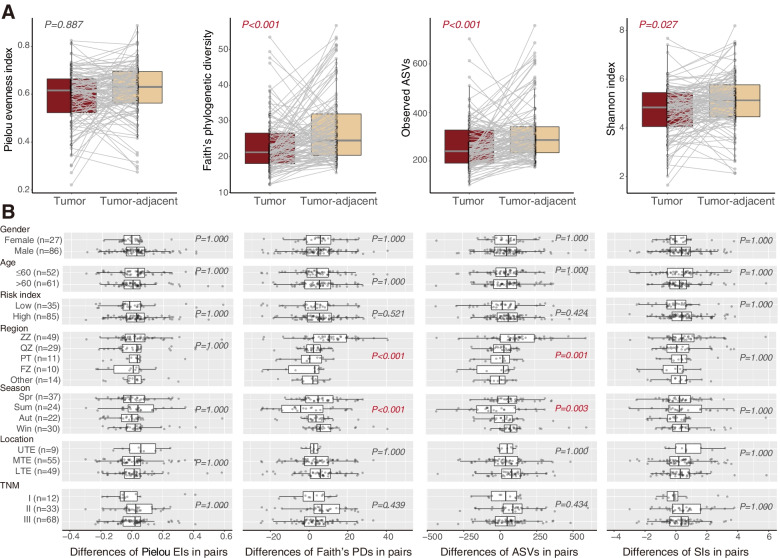
Microbial comparison for alpha diversity between tumor and tumor-adjacent tissues. **A** Alpha diversity based on the Pielou evenness index (*P* = 0.887), Faith’s phylogenetic diversity (*P* < 0.001), observed ASVs (*P* < 0.001), and Shannon index (*P* = 0.027). **B** General linear regression analysis to detect pairwise differences in alpha diversity after adjusting for gender, age, risk index, region, sampling season, tumor location, and TNM stage

For alpha diversity, four characteristic metrics were evaluated. The alpha diversity in tumor-adjacent tissue was significantly higher than that in tumor tissue, except for Pielou evenness index (Faith’s phylogenetic diversity *P* < 0.001, observed ASVs *P* < 0.001, Shannon index *P* = 0.027) (Fig. 1A). The difference in alpha diversity between paired tumor and tumor-adjacent tissue was only observed in regions (Faith’s phylogenetic diversity *P* < 0.001, observed ASVs *P* < 0.001) and sampling seasons (Faith’s phylogenetic diversity *P* < 0.001, Observed ASVs *P* = 0.003) (Fig. 1B).

**Fig. 2:**
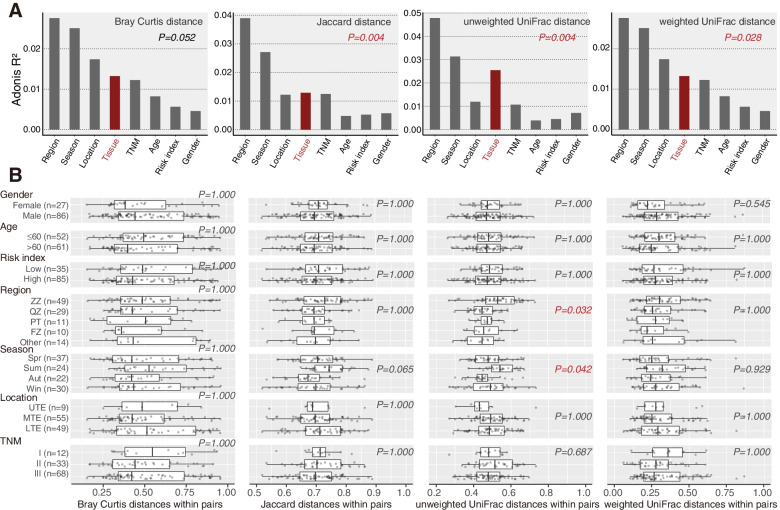
Microbial comparison for beta diversity using the multivariate Adonis test between tumor and tumor-adjacent tissues. **A** Beta diversity based on Bray Curtis distance (*P* = 0.052), Jaccard distance (*P* = 0.004), unweighted UniFrac distance (*P* = 0.004), and weighted UniFrac distance (*P* = 0.028). **B** General linear regression analysis to detect within-pair differences in beta diversity after adjusting for gender, age, risk index, region, sampling season, tumor location, and TNM stage

In the multivariate Adonis test, the beta diversity of tumor tissue was significantly different from that of tumor-adjacent tissue, as measured by Jaccard distance (*P* = 0.004), unweighted UniFrac distance (*P* = 0.004) or weighted UniFrac distance (*P* = 0.028) (Fig. 2A). About 1–2% of the variance in beta diversity was explained by tissue type. Moreover, the differences in beta diversity between within paired tumor and tumor-adjacent tissue were associated with regions (*P* = 0.032) and sampling seasons (*P* = 0.042) based on unweighted UniFrac distance (Fig. 2B).

### Microbial composition analysis

**Fig. 3:**
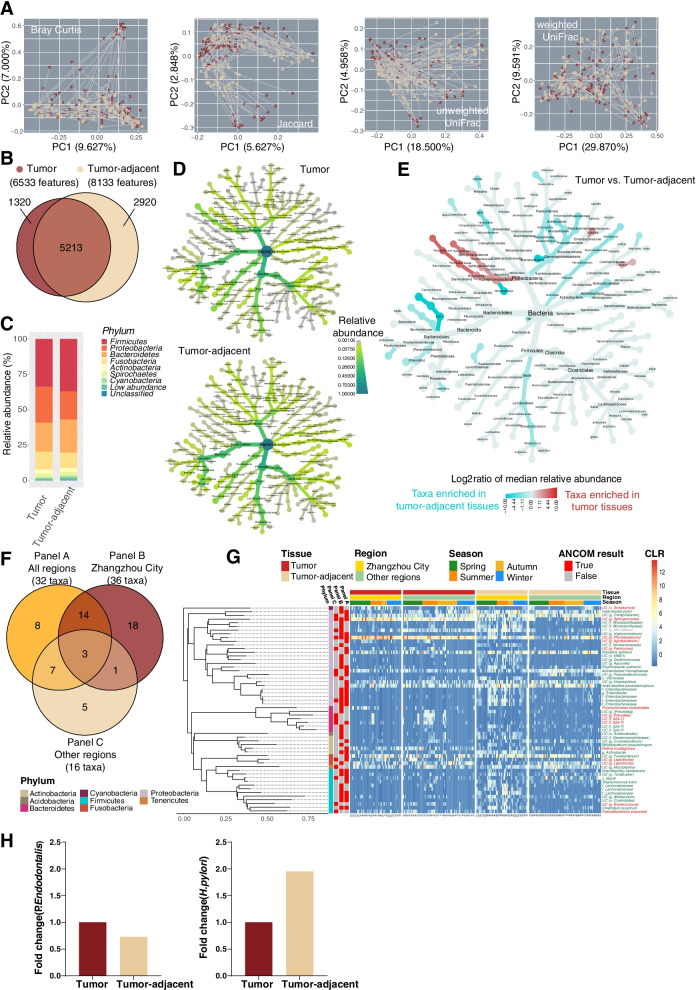
The profile of esophageal microbiota between tumor and tumor-adjacent tissues. **A** PCoA plots based on four distances between tumor and tumor-adjacent tissues. **B** The overlap of microbiota features between tumor and tumor-adjacent tissues. **C** Microbial relative abundances at the phylum level in tumor and tumor-adjacent tissues. **D** Heat tree plot of the relative abundance (higher than 0.1%) of microbiota in tumor and tumor-adjacent tissues. **E** The heat trees of microbiota for log2 ratio of median relative abundance between tumor and tumor-adjacent tissues by univariate Wilcoxon rank sum test. **F** The differentially abundant taxa between cancer and para-cancer in all regions (Panel A), Zhangzhou city (Panel B) and other regions (Panel C) after multivariate adjustment using ANCOM2. **G** Heatmap of the relative abundance of differential microbiota (the red words on the right side of the heatmap are enriched differential taxa in tumor tissue, and the green words are enriched that in tumor-adjacent tissue)

Pairwise Principal coordinate analysis (PCoA) results are displayed in Fig. 3A. Based on Bray Curtis, Jacarrd, and unweighted UniFrac distance, the microbiota from the two tissue types were separated into clusters. A total of 9453 features were found after the sequence was denoised in all samples (Supplementary file 1). More taxa were observed in tumor-adjacent tissue than in tumor tissue (8133 vs. 6533) and approximately 5213 taxa were detected in both tissues (Fig. 3B). The composition of the esophageal microbiota between tumor and tumor-adjacent tissue at the phylum and genus levels is shown in Fig. 3C and Fig. S1, respectively. To elucidate the phylogenetic relationship of the tumor and tumor-adjacent microbiota, the heat trees of microbiota (relative abundance > 0.1%) were plotted, as shown in Fig. 3D. The relative abundance of corresponding branches of bacteria in the phyla Proteobacteria, Bacteroidetes, Fusobacteria and Firmicutes was similar between tumor and tumor-adjacent tissue. However, some bacteria were enriched in different tissues (Fig. 3E). In class Alphaproteobacteria, the branches of bacteria of order Rhizobiales and Sphingomonadales were enriched in tumor tissue, while order Rhodospirillles was higher in tumor-adjacent tissue. Most bacteria of the class Alphaproteobacteria and its branch were enriched in tumor tissue, except for the family Klebsiella. Moreover, the microbiota from phylum TM7 and its branches of bacteria, class Bacilli, family Helicobacteraceae and its genus *Helicobacter* and its species *pylori* were higher in tumor-adjacent tissue.

### Differential abundance analysis

A total of 56 differential taxa were selected by the ANCOM2 algorithm (Fig. 3G). As the host region exerted the strongest effect on the microbiota, we divided all participants into the Zhangzhou group and other regions group (Table S3). There were 32, 36, and 16 differentially abundant taxa between tumor and tumor-adjacent tissue in the all regions group, the Zhangzhou group and other regions group, respectively (Fig. 3F). There were only three shared differential bacteria in tumor and tumor-adjacent tissue from different regions, named family Enterobacteriaceae, unclassified species from genus *Sphingomonas* and genus *Phyllobacterium* (Fig. 3G). It was clearly ascertained that differential bacteria in the phyla Firmicutes and Proteobacteria were enriched in tumor-adjacent tissue from Zhangzhou city. Moreover, we observed an interesting alteration in which the unclassified species in the genus *Mycoplane* was enriched in tumor tissue from other regions but enriched in tumor-adjacent tissue from Zhangzhou city. Sampling seasons were another powerful factor that influenced host microbiota (Fig. 3G). The microbiota in the phylum Cyanobacteria in tumor tissue sampled in summer had a higher relative abundance. The relative abundance of bacteria in the phylum Proteobacteria was significantly enriched in tumor-adjacent tissue when sampled in spring and summer from Zhangzhou city.

Hence, the dominant candidate differential taxa (Table 1) between tumor and tumor-adjacent tissue were selected according to the grand means of relative abundance that exceeded 0.1% from the above 56 differential taxa. They were species *R. mucilaginosa*, *P. endodontalis*, unclassified species in the genus *Leptotrichia,* unclassified species in the genus *Phyllobacterium*, and unclassified species in the genus *Sphingomonas*, which were enriched in tumor tissue. On the other hand, class Bacilli, *N. subflava*, *H. pylori*, *A. parahaemolyticus*, *A. rhizosphaerae*, unclassified species in the genus *Campylobacter* and unclassified species in the genus *Haemophilus* were enriched in tumor-adjacent tissue. Next, to explore the confounding effect of regions, sampling seasons, tumor location and risk index on host microbiota, we added these four covariates into the ANCOM2 analysis. The results showed that the relative abundance of unclassified species in the genus *Leptotrichia*, unclassified species in the genus *Sphingomonas*, and *A. rhizosphaerae* could be influenced by region; the relative abundance of unclassified species in the genus *Campylobacter* could be influenced by sampling season and tumor location. However, none of the differential taxa were significantly different in either the low- or high-risk indices in ESCC.

**Table 1:** The candidate taxa with differential abundance between tumor and tumor-adjacent tissues in ESCC^a^

Phylum	Class	Order	Family	Genus	Species	Relative abundance (%)^b^		FC^c^	ANCOM’s W (normalized W)^d^	Association with other covariates^e^			
						T	TA			Region	Season	Location	RI
*Actinobacteria*	*Actinobacteria*	*Actinomycetales*	*Micrococcaceae*	*Rothia*	*Mucilaginosa*	0.291	0.043	6.767	644 (0.850)	FALSE	FALSE	FALSE	FALSE
*Bacteroidetes*	*Bacteroidia*	*Bacteroidales*	*Porphyromonadaceae*	*Porphyromonas*	*Endodontalis*	0.286	0.027	10.593	465 (0.613)	FALSE	FALSE	FALSE	FALSE
*Firmicutes*	*Bacilli*	*–*	*–*	*–*	*–*	0.009	0.482	0.019	608 (0.802)	FALSE	FALSE	FALSE	FALSE
*Fusobacteria*	*Fusobacteriia*	*Fusobacteriales*	*Leptotrichiaceae*	*Leptotrichia*	*UC*	0.482	0.148	3.257	147 (0.194)	TRUE	FALSE	FALSE	FALSE
*Fusobacteria*	*Fusobacteriia*	*Fusobacteriales*	*Leptotrichiaceae*	*Leptotrichia*	*UC*	0.287	0.037	7.757	686 (0.905)	FALSE	FALSE	FALSE	FALSE
*Proteobacteria*	*α-Proteobacteria*	*Rhizobiales*	*Phyllobacteriaceae*	*Phyllobacterium*	*UC*	8.805	0.953	9.239	678 (0.894)	FALSE	FALSE	FALSE	FALSE
*Proteobacteria*	*α-Proteobacteria*	*Sphingomonadales*	*Sphingomonadaceae*	*Sphingomonas*	*UC*	2.736	0.412	6.641	532 (0.702)	TRUE	FALSE	FALSE	FALSE
*Proteobacteria*	*β-Proteobacteria*	*Neisseriales*	*Neisseriaceae*	*Neisseria*	*Subflava*	0.458	0.950	0.482	608 (0.802)	FALSE	FALSE	FALSE	FALSE
*Proteobacteria*	*ε-Proteobacteria*	*Campylobacterales*	*Campylobacteraceae*	*Campylobacter*	*UC*	0.253	0.555	0.456	686 (0.905)	FALSE	TRUE	TRUE	FALSE
*Proteobacteria*	*ε-Proteobacteria*	*Campylobacterales*	*Helicobacteraceae*	*Helicobacter*	*Pylori*	0.322	0.904	0.356	732 (0.966)	FALSE	FALSE	FALSE	FALSE
*Proteobacteria*	*γ-Proteobacteria*	*Pasteurellales*	*Pasteurellaceae*	*Actinobacillus*	*Parahaemolyticus*	0.685	1.452	0.472	678 (0.894)	FALSE	FALSE	FALSE	FALSE
*Proteobacteria*	*γ-Proteobacteria*	*Pasteurellales*	*Pasteurellaceae*	*Haemophilus*	*UC*	0.068	0.171	0.398	532 (0.702)	FALSE	FALSE	FALSE	FALSE
*Proteobacteria*	*γ-Proteobacteria*	*Pseudomonadales*	*Moraxellaceae*	*Acinetobacter*	*Rhizosphaerae*	0.057	0.233	0.245	644 (0.850)	TRUE	FALSE	FALSE	FALSE

*UC* unclassified, *T* tumor tissue, *TA* tumor-adjacent tissue, *FC* fold change, *RI* risk index

**Fig. 4:**
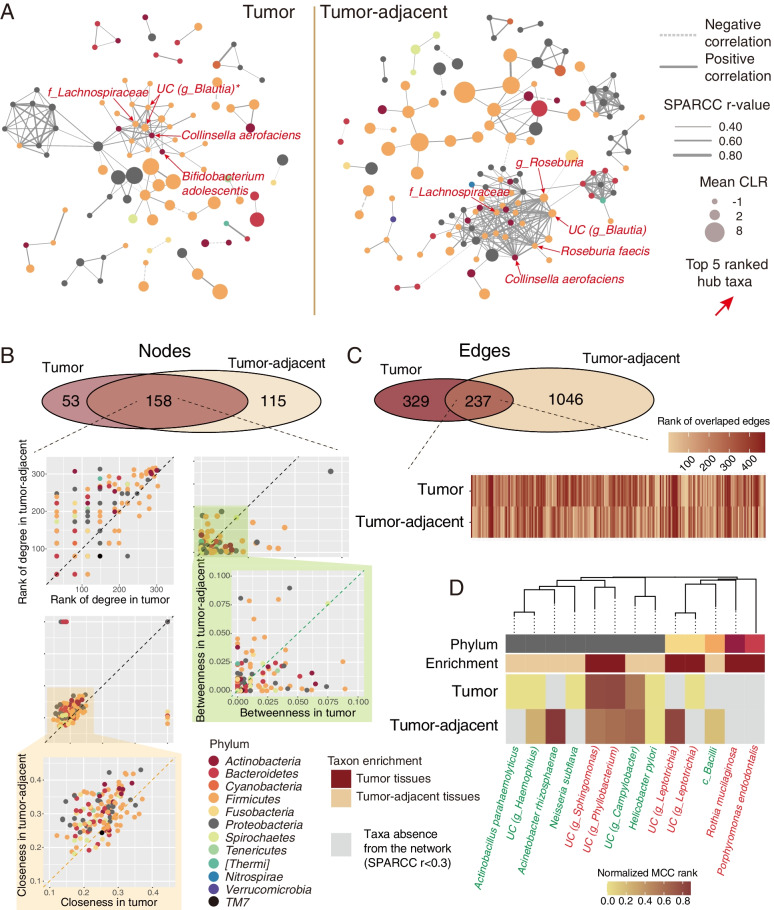
The analyses of microbial co-occurrence networks. **A** Co-occurrence network of the microbiota in tumor and tumor-adjacent tissue (only Sparcc absolute r > =0.4 is shown). **B** Discrepancies of co-occurrence network nodes between tumor and tumor-adjacent tissue and its measurement dimensions included degree, betweenness, and closeness centrality. **C** Discrepancies of co-occurrence edges between tumor and tumor-adjacent tissues. **D** Discrepancies in the importance of differential taxa in the co-occurrence network

^a^ The candidate differential taxa were selected according to the following two conditions: 1) pass the ANCOM tests conducted in patients from Zhangzhou City (50 pairs) or the other regions (70 pairs), or the pooled population (120 pairs) (Fig. 4G); 2) the grand means of relative abundance were exceeded 0.1%

^b^ Mean relative abundance were presented

^c^ Fold change (FC) = mean relative abundance in tumor/ mean relative abundance in tumor-adjacent tissue

^d^ The differential abundant taxa between tumor and tumor-adjacent tissues were selected by the ANCOM2 algorithm under detected cut-off at 0.7, and were adjusted for sex, age, risk index, TNM, season, tumor location and regions. The normalized ANCOM’s W statistics were calculated by divided the W over the number of total taxa which were identified as none-structural zero

^e^ The ANCOM2 algorithm was applied for association detection. The detected cut-off at 0.7 was adopt for all analyses. The variables included in ANCOM2 comparisons were in line with those included in differential abundant taxa detection. TRUE or FALSE indicated that the relative abundance of candidate taxa could be or not be influenced by specific factors, respectively

### qPCR validation of esophageal species differential abundance

To validate the species we identified as differentially abundant in ESCC from our 16S rRNA-based microbiome analyses, we used qPCR to quantify the presence of *P. endodontalis* and *H. pylori*. Fold changes of qPCR abundances in tumor versus tumor-adjacent tissue are shown in Fig. 3H. We measured an increase for *P. endodontalis* (*P* = 0.320) and a decrease for *H. pylori* (*P* = 0.160) in tumor-adjacent samples, demonstrating consistency with the result of the altered abundance of these species based on 16S rRNA-based analyses.

### Microbial co-occurrence networks

To understand the interaction among esophageal microbiota in tumor and tumor-adjacent tissue, we illustrated the microbial co-occurrence networks of the two groups. There were 3089 positive and 348 negative correlations in the tumor tissue, while the tumor-adjacent samples had 3761 positive and 355 negative correlations. Obviously, the microbial co-occurrence networks were distinct between the tumor and tumor-adjacent tissue (Fig. 4A). However, wide correlations were investigated in the family Lachnospiraceae, species *C. aerofaciens* and unclassified species in the genus *Blautia* in both tissue types.

To quantify such differences, we counted the number of nodes and their centrality in the microbial networks in different tissue types. As expected, the numbers of interacting microbiota constituents in tumor-adjacent tissue (273 nodes) were higher than those in tumor tissue (201 nodes) (Fig. 4A and B). The centrality of co-occurrence networks was described with three dimensions: degree, betweenness, and closeness centrality. Interestingly, the degree and closeness of shared nodes between tumor and tumor-adjacent tissue were quite different. Next, the edges of the networks were evaluated (Fig. 4C). Despite having a few overlapping edges, the distribution of the rank of overlapping edges varies in the tumor and tumor-adjacent tissue.

To show the importance of the above candidate differential taxa in the network, the heatmap is shown in Fig. 4D. Most of the differentially abundant taxa in the network were from the phylum Proteobacteria, and certain differentially abundant taxa did not emerge in the co-occurrence networks. The roles of differential taxa in different networks (tumor vs. tumor-adjacent tissue) also varied. The discrepancies in microbial co-occurrence networks in the two groups may be attributed to the specific metabolism in different tissue types.

### The association between esophageal microbiota and predicted function

**Fig. 5:**
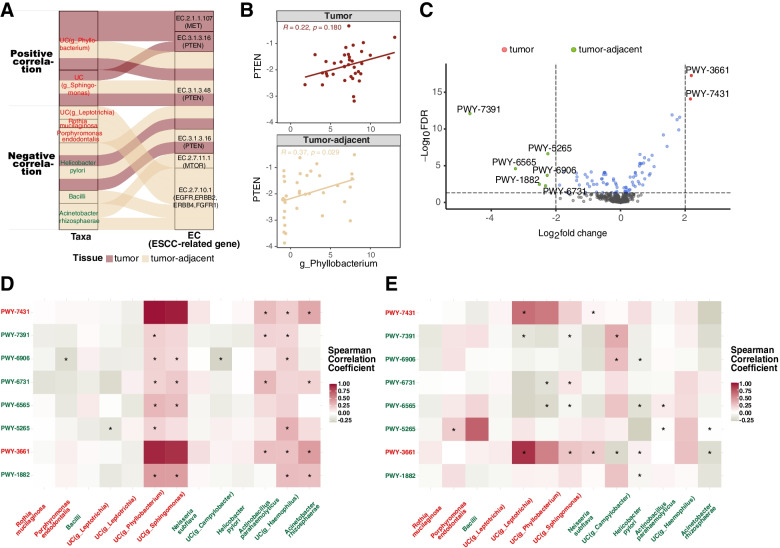
The association between esophageal microbiota and predicted function. **A** The association between the differential microbiota and ESCC-related functional enzymes. **B** The correlation between the expression of PTEN by qRT-PCR and the abundance of unclassified species in the genus *Phyllobacterium.* **C** Volcano plot shows the differential MetaCyc metabolic pathways between tumor and tumor-adjacent tissues. The X-axis indicates the log_2_(fold change), and the Y-axis indicates -log_10_(FDR). The significant pathways enriched in tumor tissue (FDR < 0.05 and log_2_ fold change> 2) are colored as red dots, that are increased in tumor-adjacent tissue (FDR < 0.05 and log_2_ fold change<− 2) are colored as green dots. **D**, **E** The heatmap indicates the association between the differential MetaCyc metabolic pathways and microbiota in tumor tissue (**D**) and tumor-adjacent tissue (**E**)

Most of the differentially abundant taxa in tumor-adjacent tissue were negatively associated with EC 2.7.10.1, which regulated the epidermal growth factor receptor (EGFR), erb-b2 receptor tyrosine kinase 2 (ERBB2), erb-b2 receptor tyrosine kinase 4 (ERBB4), and fibroblast growth factor receptor 1 (FGFR1) signaling pathways. In contrast, the differentially abundant taxa in tumor were positively associated with EC2.1.1.107 and EC3.1.3.16 in the MET proto-oncogene, receptor tyrosine kinase (MET) and PTEN signaling pathways respectively (Fig. 5A). Moreover, the expression of PTEN by qRT-PCR was positively correlated with the abundance of unclassified species in genus *Phyllobacterium* in both tumor (*R* = 0.22, *P* = 0.180) and tumor-adjacent tissues (*R* = 0.37, *P* = 0.029), which was in line with the predicted results (Fig. 5B). Among the eight MetaCyc metabolic pathways that were significantly different between tumor and tumor-adjacent tissues (Fig. 5C, Table S4), the relative abundance of PWY-3661 and PWY-7431 was increased in the tumor tissue, and six other pathways (PWY-1882, PWY-5265, PWY-6565, PWY-6731, PWY-6906, and PWY-7391) were enriched in tumor-adjacent tissue. Notably, the differentially abundant taxa played various roles in different tissues (Fig. 5D-E). For example, unclassified species from the genera *Phyllobacterium* and *Sphingomonas* were positively associated with PWY-6906 and PWY-1882 in tumors but negatively with PWY-6731 and PWY-6565 in tumor-adjacent tissue. Similarly, the correlation was demonstrated between taxa that were *A. parahaemolyticus* and *A. rhizosphaerae* and pathways including PWY-7431, PWY-7391, PWY-6731, and PWY-1882 in tumor tissue, but PWY-6565 in tumor-adjacent tissue. In addition, the unclassified species in the genus *Haemophilus* was only associated with the pathways in tumors.

## Discussion

To characterize the esophageal microbiota in ESCC, we compared the microbial diversity and composition of paired tumor and tumor-adjacent tissues for ESCC in this study. Moreover, 56 candidate differential taxa were identified, of which 13 taxa were more abundant. In addition, we suggested that the microbial co-occurrence network was another critical aspect of the microbial community. Furthermore, the relationship between differential taxa and predicted functional ESCC-related gene pathways was investigated.

We observed differences in microbial diversity between the tumor and tumor-adjacent tissues for ESCC, which was similar to the findings of Yang [8] and Yu [9]. The microbial structure is modified due to the altered esophageal microenvironment in the progression of carcinogenesis. Interestingly, in the general linear regression model, the within-pair differences in microbial diversity were only impacted by regions and sampling seasons, which was possibly attributed to regions and sampling seasons explaining most of the observed variations in the diversity measures of esophageal microbiota (Fig. 2A). This is in agreement with many studies that environmental factors including sampling regions or seasons are the relevant factors affecting microbiota [13, 25]. In addition, the microbiota composition varies in response to other factors [26], such as age, sex, diet, nutritional status, environmental factors, lifestyles, and medications. According to Gupta [27], the important role of regions and seasons in microbiota diversity has been attributed to differences in host genetics or other host-specific factors such as innate/adaptive immunity, diet, and environmental exposure. Therefore, further studies involving microbiomics are needed to consider the effect of these factors on the microbiota to ensure generalizability.

Several differential bacteria were detected in tumor-adjacent tissue for ESCC. Of note, it has been reported that *R. mucilaginosa* upregulated TNF-α, and the upregulation of CD36 in all cell lines in cocultures [28] and the increased abundance of *R. mucilaginosa* exhibits the ability to produce acetaldehyde [29, 30]. The above results could explain the findings of the present study of a higher *R. mucilaginosa* in esophageal tumor tissue than that in tumor-adjacent tissue. In accordance with our results, Richard et al. [31] and Liu [32] identified that the tumor mucosal genus *Sphingomonas* had a particularly high prevalence in colitis-associated and bladder cancer. If the microbiota is involved in carcinogenesis, we can expect that it will be enriched in the tumor-adjacent tissue according to Wang’s hypothesis [33]. Many studies have indicated *N. subflava* is significantly more abundant in healthy controls, as found in our study [34, 35]. In addition, similar observations previously reported that *H. pylori* was increased in adjacent compared to tumor tissues for gastric cancer, which is in line with our results [36, 37]. Sandra investigated *Campylobacter* species that appear to be more prevalent and abundant in the esophagus of patients in Barrett’s esophagus [38], but their abundance decreased with the progression to EAC [39]. Our current study proved this finding in ESCC. Furthermore, we used species-specific qPCR to validate our 16S rRNA-based findings of enrichment of *P. endodontalis* and reduction of *H. pylori* in tumor-adjacent tissue, thus providing confidence for our results. At present, there is no other study of esophageal microbiota in ESCC that has taken this additional step of qPCR to corroborate 16S rRNA-based results.

A disease-associated microenvironment could be affected by a specific microbial network. Several studies have shown ecological interaction networks of microbiota in tumor and adjacent mucosal tissues in gastric [37] and colorectal cancer [33]. The esophagus is a part of the gastrointestinal tract, and we highlight the esophageal microbial co-occurrence network for ESCC to extend digestive research. Our microbial co-occurrence network analysis suggests that bacterial compositions in different sample types show specific correlation patterns. Interestingly, strong co-occurrence interactions formed by the family Lachnospraceae, *C. aerofaciens*, and undefined species from the genus *Blautia* showed the centralities of these taxa in both networks. This suggests that hub taxa may have a predominant impact on the structure of the microbiota in ESCC patients, which deserves further investigation. Notably, the above differentially abundant taxa also participated in the co-occurrence network and had various degrees of importance. It is implied that the microbial interaction is complex, and full consideration of the microbial interaction network should be taken in further microbiome studies.

We have preliminarily revealed differences in the predicted microbiota functions in tumor and tumor-adjacent tissues. Interestingly, regardless of the presence or absence of mutations in these genes in ESCC, their products (ERBB2 and MET) are often overexpressed in tumor tissues, indicating their vital carcinogenic role in ESCC [22]. It is possible that the abnormal profiled abundance of differential bacteria causes changes in the expression of PTEN, ERBB2, ERBB4, and MET and stimulates tumor growth. Additionally, the enriched differential MetaCyc pathways in tumors are linked to amino acid degradation. It has been implicated that microbiota in dysplasia tissues perturbed amino acid metabolism that is probably involved in the process of tumor development [40]. On the other hand, a large proportion of increased pathways in tumor-adjacent tissue are associated with bacteria-related biomolecule synthesis. This could explain why the more abundant microbiota were detected in tumor-adjacent samples. We found that *H. pylori* was negatively associated with polyamine biosynthesis, which was in line with Takashima [41], who found investigation that *H. pylori* significantly inhibited the proliferation of polyamine biosynthesis enzymes in cells. Moreover, Rousseau [42] found that *Haemophilus* in animals created changes in peptidoglycan metabolism, which is also consistent with our results. To develop a deeper understanding of esophageal carcinogenesis, further studies are needed to examine the significance of microbial functional variations in the ESCC microenvironment.

Association-based research provides limited insight into the role of microbiota in cancer. The microbial community in patients with cancer interacts with their hosts and influences tumor development by a variety of mechanisms. Unraveling the causal relationships between microbiota and cancer, as well as understanding the underlying mechanisms, has become the focus of future research.

In this study, esophageal mucosa samples were obtained from ESCC patients undergoing surgery, thus avoiding possible oral microbial contamination that may occur during upper digestive endoscopy sampling. Our work provides insights into the composition, function and interaction network of the mucosa-associated bacterial community in the tumor microenvironment in ESCC. However, our study had certain limitations. First, our experimental protocol for extracting the DNA of bacteria did not include a bead-beating step. This could lead to an overestimation of the proportion of Gram-negative bacteria in the results. Additionally, this study did not include esophageal tissues from individuals without ESCC for comparison, and the application of these results is limited. In addition, 16S rRNA sequencing only amplified the 16S rRNA-specific region and was less sophisticated than metagenomic sequencing. This is another limitation of the current study.

## Conclusion

Compared with tumor-adjacent tissues, the microbiota in tumor tissues showed significant differences in diversity and composition. Alterations in the microbial co-occurrence network and functional pathways in ESCC tissues may be involved in carcinogenesis and the maintenance of the local microenvironment for ESCC. These discoveries of the esophageal microbiota for ESCC patients may contribute to the etiology for ESCC prevention, diagnosis, early intervention, and treatment.

## Supplementary Information

**Additional file 1: Supplementary File 1.** The details of method

**Additional file 2: Supplementary File 2.** Risk index of esophageal squamous cell carcinoma (ESCC)

**Additional file 3: Table S1.** The relative abundance of 955 features in ESCC tumor and tumor-adjacent tissues.

**Additional file 4: Table S2.** Basic information of 120 ESCCs.

**Additional file 5: Table S3.** The characteristics of ESCC patients from Zhangzhou City (*n* = 50) and other regions (*n* = 70).

**Additional file 6: Table S4.** The corresponding description of the differential MetaCyc metabolic pathways.

**Additional file 7: Fig. S1.** Microbial relative abundances at the genus level in tumor and tumor-adjacent tissues.

## Data Availability

The datasets analysed during the current study are available from the corresponding author on reasonable request.

## Abbreviations

ESCC: Esophageal squamous cell carcinoma
EAC: Esophageal adenocarcinoma
HLN: Harvested lymph nodes
TNM: Tumor, node, and metastasis
HE: Haematoxylin-eosin
SDS: Sodium dodecyl sulfate
QIIME: Quantitative insights into microbial ecology
ASVs: Amplicon sequence variants
FDR: False discovery rate
PICRUSt2: Phylogenetic investigation of communities by reconstruction of unobserved states 2
qPCR: Quantitative PCR
Ct: Cycle threshold
qRT-PCR: quantitative reverse transcription PCR
ANCOM2: Analysis of composition of microbiomes 2
KEGG: Kyoto Encyclopedia of Genes and Genomes
EGFR: Epidermal growth factor receptor;
ERBB2: Erb-b2 receptor tyrosine kinase 2
ERBB4: Erb-b2 receptor tyrosine kinase 4
FGFR1: Fibroblast growth factor receptor 1
MET: MET proto-oncogene, receptor tyrosine kinase
PTEN: Phosphatase and tensin homolog
GAPDH: Glyceraldehyde-3-phosphate dehydrogenase
