# Supplementary material for: Characteristics and interplay of esophageal microbiota in esophageal squamous cell carcinoma
Source: BMC Cancer. 2022 Jun 24;22:696. doi: 10.1186/s12885-022-09771-2 (PMC9229141; doi:10.1186/s12885-022-09771-2)
Supplement: Supplementary file 1 — Additional file 1: Supplementary File 1. The details of method [file 12885_2022_9771_MOESM1_ESM.docx]

**Supplementary File 1. The details of method**

**Studying population**

We performed a hospital-based retrospective study of 120 patients pathologically diagnosed with primary ESCC between February 2013 and October 2017 at Fujian Provincial Cancer Hospital and Zhangzhou Municipal Hospital(**Figure A**).

**Sequence data processing**

Raw sequencing data from patients with ESCC were imported into Quantitative Insights Into Microbial Ecology (QIIME2-2020.02) [1] and processed using the DEBLUR algorithm to denoise and then inferred exact amplicon sequence variants (ASVs). The detailed analysis workflow was presented in **Figure B**. The curated ASVs were aligned and annotated by the Naïve Bayes classifier using the Greengenes (version 13.5) database, and were used for the subsequent construction of the phylogenetic tree. ASVs were submitted to a pre-trained Naïve Bayes Classifier (NBC) trained on full-length 99% Greengenes reference for the taxonomic classification. Before diversity analysis, the threshold for rarefaction depth was decided by minimizing sequences loss while maximizing the number of samples. At the depth of 10000 sequences per sample, the richness of observed communities had tended to be saturated, with 113 pairs of cancer and para-cancer samples were kept for the Alpha and Beta diversity metrics construction.

**Statistical analysis**

Questionnaires and clinicopathological data were double-entered into EpiData (version 3.1, Denmark). The demographic and baseline clinical features were displayed using n(%).The individuals’ risk index of ESCC was calculated by variables included age, smoking, drinking, eating speed, hot food, pickled food, and fruit from questionnaire (**Supplementary file 2**). All statistical analyses were evaluated using R software (R version 4.0.2), and two-tailed *P*<0.050 was considered statistically significant.

Since the samples were paired, the Wilcoxon sign rank test was applied for comparisons of Alpha diversity (observed ASVs, Shannon index, and Faith’s Phylogenetic Diversity) between cancer and matched para-cancer tissues. For Beta diversity (Bray-Curtis, Jacarrd, unweighted UniFrac and weighted UniFrac distances), the Adonis action of PERMANOVA tests were implemented to evaluate whether the variation of distances could be explained by other controlled variables (ESCC risk scores, sampling seasons, residential regions, tumor locations, and TNM stages).

The general linear models were used to test whether the aforementioned controlled variables could impact the diversity metrics. For Alpha diversity, the paired differences of diversity metrics within each paired sample were calculated by subtracting the diversity values from cancer tissues to the corresponding para-cancer tissues and served as dependent variables. While for the Beta diversity, the dependent variables were defined as the pairwise distances between cancer and matched para-cancer tissues (within-subject distances). Before linear regression, all dependent variables were checked for normality, and the natural logarithmic transformation was applied for reducing skewness. All *P* values for controlled variables were corrected by the Benjamini-Hochberg FDR procedure.

PCoA plotting which based on the Bray-Curtis, Jacarrd, unweighted UniFrac and weighted UniFrac distances were used to depict the microbiome composition. The ANCOM2 tests [2] were performed to detect the differential abundance in different tissue groups. It had been documented that the regional variation limits applications of microbial-disease association models, therefore, the data were split into Zhangzhou group (50 pairs of samples) and other Cities from Fujian Province group (70 pairs of tissues) according to patients’ residential regions.

For the functional prediction, the PICRUSt2 [3] pipeline was used to generate predictions for EC numbers and MetaCyc pathways. The strength of edges of microbial co-occurrence network were assessed by SPARCC algorithm [4], and the interaction network diagram visualized with Cytoscape [5]. The top hub taxa were assessed by plugin cytoHubba [6] in Cytoscape. Then, we selected four well established pathogenesis enzyme genes in ESCC according to Lin [7] from KEGG database. Then, Spearman correlation was performed to explore the association between the differential taxa and four enzyme genes. DESeq algorithm [8] was applied to calculate the differential MetaCyc pathways and visualized by volcano plot, then the Spearman correlation between differential taxa and differential MetaCyc pathways was calculated.


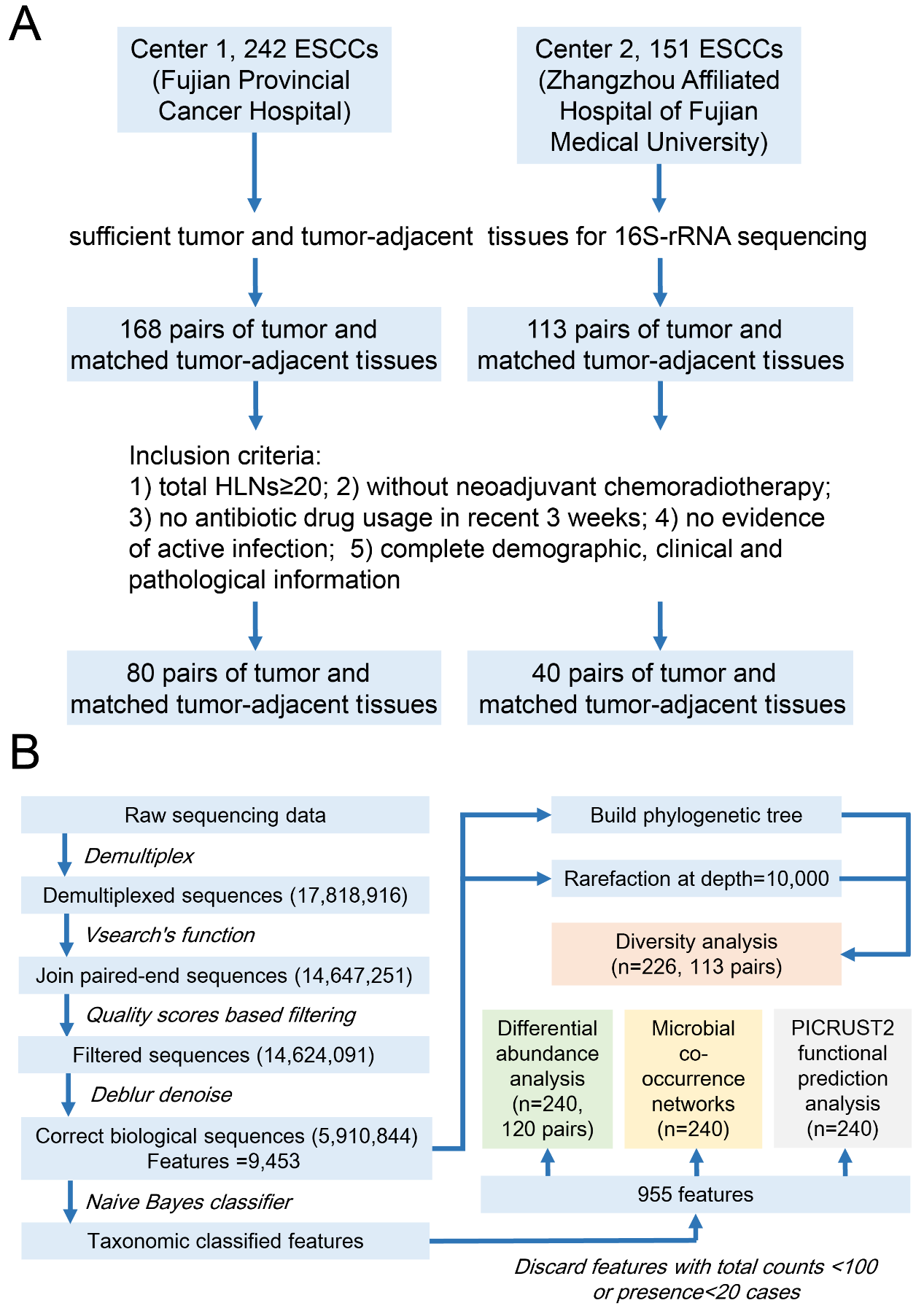


**Figure.** Flowcharts of inclusion criteria of participants and process of sequencing data. (A) Inclusion procedures for participants with ESCC. (B) Analysis workflow for sequencing data.

**References:**

1. Bolyen E, Rideout JR, Dillon MR, Bokulich NA, Abnet CC, Al-Ghalith GA et al. Reproducible, interactive, scalable and extensible microbiome data science using QIIME 2. Nat Biotechnol 2019; 37(8):852–7.

2. Mandal S, van Treuren W, White RA, Eggesbø M, Knight R, Peddada SD. Analysis of composition of microbiomes: a novel method for studying microbial composition. Microb Ecol Health Dis 2015; 26:27663.

3. Douglas GM, Maffei VJ, Zaneveld JR, Yurgel SN, Brown JR, Taylor CM et al. PICRUSt2 for prediction of metagenome functions. Nat Biotechnol 2020; 38(6):685–8.

4. Friedman J, Alm EJ. Inferring correlation networks from genomic survey data. PLoS computational biology 2012; 8(9):e1002687.

5. Smoot ME, Ono K, Ruscheinski J, Wang P-L, Ideker T. Cytoscape 2.8: new features for data integration and network visualization. Bioinformatics 2011; 27(3):431–2.

6. Chin C-H, Chen S-H, Wu H-H, Ho C-W, Ko M-T, Lin C-Y. cytoHubba: identifying hub objects and sub-networks from complex interactome. BMC Syst Biol 2014; 8 Suppl 4:S11.

7. Lin D-C, Wang M-R, Koeffler HP. Genomic and Epigenomic Aberrations in Esophageal Squamous Cell Carcinoma and Implications for Patients. Gastroenterology 2018; 154(2):374–89.

8. Love MI, Huber W, Anders S. Moderated estimation of fold change and dispersion for RNA-seq data with DESeq2. Genome Biol 2014; 15(12):550.
