# Supplementary material for: Characteristics and interplay of esophageal microbiota in esophageal squamous cell carcinoma
Source: BMC Cancer. 2022 Jun 24;22:696. doi: 10.1186/s12885-022-09771-2 (PMC9229141; doi:10.1186/s12885-022-09771-2)
Supplement: Supplementary file 2 — Additional file 2: Supplementary File 2. Risk index of esophageal squamous cell carcinoma (ESCC) [file 12885_2022_9771_MOESM2_ESM.docx]

**Supplementary File 2. Risk index of esophageal squamous cell carcinoma (ESCC)**

A hospital-based case-control study was conducted for the ESCC risk index construction. We included 1173 diagnosed ESCC cases and 604 controls which had been reported in our previous researches [1-5]. The information of demographics (gender and age), lifestyle exposure (tobacco smoking, alcohol and tea drinking) and dietary habits (eating speed, the preference of hot, hard, pickled and fried food, as well as the frequencies of fresh fruit and vegetable intake) were collected by using structured epidemiological questionnaires [4, 5].

In order to simplify the computation and to enhance the interpretability, all variables were re-coded into binary categories, with high values indicated higher ESCC risk. By logistic regression which included all factors listed in Table-S1, the individual risk index was synthesized by summing the products of significant variables values and corresponding multivariable-adjusted regression coefficients [4, 6].

Table-S1. The characteristics of the study population

and the risk index coefficients for ESCC

| Variables | Controls  [n (%)] | Cases  [n (%)] | *β* | *OR* (95% *CI*) | *P* | Coefficients |
| --- | --- | --- | --- | --- | --- | --- |
| Gender |  |  |  |  |  |  |
| Female | 232 (38.4) | 309 (26.3) | - |  |  |  |
| Male | 372 (61.6) | 864 (73.7) | -0.241 | 0.786 (0.561, 1.101) | 0.786 | - |
| Age |  |  |  |  |  |  |
| <60 | 294 (48.7) | 571 (48.7) | - |  |  |  |
| ≥60 | 310 (51.3) | 602 (51.3) | 0.231 | 1.260 (1.104, 1.566) | 0.037 | 0.231 |
| Smoking |  |  |  |  |  |  |
| No | 316 (52.3) | 387 (33.0) | - |  |  |  |
| Yes | 288 (47.7) | 786 (67.0) | 0.547 | 1.727 (1.261, 2.366) | 0.001 | 0.547 |
| Drinking |  |  |  |  |  |  |
| No | 434 (71.9) | 618 (52.7) | - |  |  |  |
| Yes | 170 (28.1) | 555 (47.3) | 0.607 | 1.834 (1.420, 2.370) | <0.001 | 0.607 |
| Tea |  |  |  |  |  |  |
| No | 187 (31.0) | 302 (25.7) | - |  |  |  |
| Yes | 417 (69.0) | 871 (74.3) | 0.128 | 1.136 (0.880, 1.467) | 0.327 | - |
| Eating speed |  |  |  |  |  |  |
| >10 min | 408 (67.5) | 639 (54.5) | - |  |  |  |
| ≤10 min | 196 (32.5) | 534 (45.5) | 0.353 | 1.423 (1.136, 1.781) | 0.002 | 0.353 |
| Hot food |  |  |  |  |  |  |
| No | 347 (57.5) | 415 (35.4) | - |  |  |  |
| Yes | 257 (42.5) | 758 (64.6) | 0.742 | 2.101 (1.692, 2.609) | <0.001 | 0.742 |
| Hard food |  |  |  |  |  |  |
| No | 304 (50.3) | 427 (36.4) | - |  |  |  |
| Yes | 300 (49.7) | 746 (63.6) | 0.338 | 1.402 (1.124, 1.749) | 0.003 | 0.338 |
| Pickled food |  |  |  |  |  |  |
| <1/week | 310 (51.3) | 510 (43.5) | - |  |  |  |
| ≥1/week | 294 (48.7) | 663 (56.5) | 0.254 | 1.289 (1.038, 1.602) | 0.022 | 0.254 |
| Fired food |  |  |  |  |  |  |
| <1/week | 433 (71.7) | 822 (70.1) | - |  |  |  |
| ≥1/week | 171 (28.3) | 351 (29.9) | -0.085 | 0.919 (0.721, 1.171) | 0.493 | - |
| Fruit |  |  |  |  |  |  |
| ≥1/week | 380 (62.9) | 489 (41.7) | - |  |  |  |
| <1/week | 224 (37.1) | 684 (58.3) | 0.744 | 2.105 (1.695, 2.614) | <0.001 | 0.744 |
| Vegetable |  |  |  |  |  |  |
| ≥1/day | 571 (94.5) | 1087 (92.4) | - |  |  |  |
| <1/day | 33 (5.5) | 89 (7.6) | 0.065 | 1.068 (0.684, 1.666) | 0.774 | - |

Therefore, the risk index (RI) for any participants could be expressed by following formula:

RI=0.231*age+0.547*smoking+0.607*dinking+0.353*eating_speed+0.742*hot_food+0.338*hard_food+0.254*pickled_food+0.744*fruit

The risk indexes (RI) of two groups were listed in Table-S2. Mann-Whitney U test indicated that the RIs in ESCC were significantly higher than those in controls (*P*<0.001, Figure-S1A), and the area (AUC) under the receiver operating characteristic curve (ROC) also showed that RI had acceptable discernibility (AUC=0.715, 95% *CI*: 0.690-0.739, Figure-S1B).

Table-S2. The risk indexes (RI) of ESCC cases and controls

| Groups | Median | (P_25_, P_75_) |
| --- | --- | --- |
| ESCC | 2.235 | (1.582, 2.856) |
| Control | 1.522 | (0.975, 2.112) |


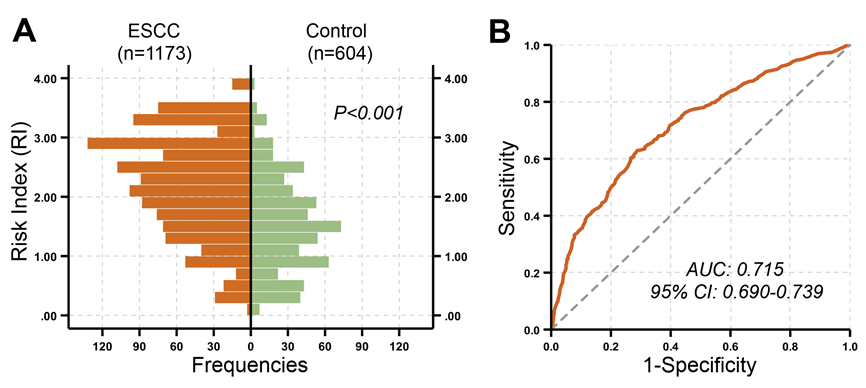


Figure-S1. The distribution and distinguishability of risk indexes (RIs). (A) The pyramid plot showed that the RIs were distributed differently between two groups. The distributions were compared across groups by Mann-Whitney U test. (B) ROC analysis was used to describe diagnostic testing performance of RI.

**References**

[1] Lin Z, Chen W, Chen Y, Peng X, Zhu K, Lin Y, et al. A new classification of lymph node metastases according to the lymph node stations for predicting prognosis in surgical patients with esophageal squamous cell carcinoma. Oncotarget. 2016;7(46):76261‐76273.

[2] Liu Y, Lin Z, Lin Y, Chen Y, Peng XE, He F, et al. Streptococcus and Prevotella are associated with the prognosis of oesophageal squamous cell carcinoma. J Med Microbiol. 2018;67(8):1058‐1068.

[3] Lin Z, Chen W, Chen Y, Peng X, Yan S, He F, et al. Achieving adequate lymph node dissection in treating esophageal squamous cell carcinomas by radical lymphadenectomy: Beyond the scope of numbers of harvested lymph nodes. Oncol Lett. 2019;18(2):1617‐1630.

[4] Liu S, Lin Z, Huang L, Chen H, Liu Y, He F, et al. Oolong tea consumption and its interactions with a novel composite index on esophageal squamous cell carcinoma. BMC Complement Altern Med. 2019;19(1):358. Published 2019 Dec 10.

[5] Yang H, Lin Z, Lin Y, He F, Liu S, Zhang Z, et al. Risk Factors of Esophageal Squamous Cell Cancer Specific for Different Macroscopic Types. Nutr Cancer. 2020;72(8):1336-1344.

[6] Balkau B, Hu G, Qiao Q, Tuomilehto J, Borch-Johnsen K, Pyörälä K, et al. Prediction of the risk of cardiovascular mortality using a score that includes glucose as a risk factor. The DECODE Study. Diabetologia. 2004;47(12):2118‐2128.
