# Supplementary material for: Characteristics and interplay of esophageal microbiota in esophageal squamous cell carcinoma
Source: BMC Cancer. 2022 Jun 24;22:696. doi: 10.1186/s12885-022-09771-2 (PMC9229141; doi:10.1186/s12885-022-09771-2)
Supplement: Supplementary file 4 — Additional file 4: Table S2. Basic information of 120 ESCCs. [file 12885_2022_9771_MOESM4_ESM.docx]

Table S2. Basic information of 120 ESCCs

| Variables | n | (%) |
| --- | --- | --- |
| Gender |  |  |
| Female | 31 | (25.83) |
| Male | 89 | (74.17) |
| Age | *61 (56, 65)*^a^ | |
| ≤60 | 56 | (46.67) |
| >60 | 64 | (53.33) |
| Region |  |  |
| Zhangzhou | 50 | (41.67) |
| Fuzhou | 11 | (9.17) |
| Putian | 11 | (9.17) |
| Quanzhou | 33 | (27.50) |
| Others | 15 | (12.50) |
| Season |  |  |
| Spring | 39 | (32.50) |
| Summer | 26 | (21.67) |
| Autumn | 24 | (20.00) |
| Winter | 31 | (25.83) |
| Tumor location |  |  |
| Upper thoracic | 11 | (9.17) |
| Middle thoracic | 57 | (47.50) |
| Lower thoracic | 52 | (43.33) |
| TNM |  |  |
| Stage I | 12 | (10.00) |
| Stage II | 36 | (30.00) |
| Stage III | 72 | (60.00) |
| Risk index^b^ | *1.99 (1.44, 2.49)*^a^ | |
| Low | 35 | (29.17) |
| High | 85 | (70.83) |

^a^ The italic numbers were the medians, 25^th^ and 75^th^ percentiles, respectively.

^b^ The median value of risk index in controls (see supplementary file 2) were used as the cut-off for low and high categories definition.
