# Supplementary material for: Characteristics and interplay of esophageal microbiota in esophageal squamous cell carcinoma
Source: BMC Cancer. 2022 Jun 24;22:696. doi: 10.1186/s12885-022-09771-2 (PMC9229141; doi:10.1186/s12885-022-09771-2)
Supplement: Supplementary file 5 — Additional file 5: Table S3. The characteristics of ESCC patients from Zhangzhou City (n = 50) and other regions (n = 70). [file 12885_2022_9771_MOESM5_ESM.docx]

Table S3. The characteristics of ESCC patients from Zhangzhou City (n=50) and other regions (n=70).

| Variables | Zhangzhou City | |  | Other regions | | *P-value*^†^ |  |
| --- | --- | --- | --- | --- | --- | --- | --- |
|  | n | (%) |  | n | (%) |  |  |
| Gender |  |  |  |  |  | *1.000* |  |
| Female | 13 | (26.0) |  | 18 | (25.7) |  |  |
| Male | 37 | (74.0) |  | 52 | (74.3) |  |  |
| Age | *62 (58, 65)^*^* | |  | *61 (55, 65)^*^* | | *0.381* |  |
| ≤60 | 21 | (42.0) |  | 35 | (50.0) |  |  |
| >60 | 29 | (58.0) |  | 35 | (50.0) |  |  |
| Season |  |  |  |  |  | *0.107* |  |
| Spring | 20 | (40.0) |  | 19 | (27.1) |  |  |
| Summer | 11 | (22.0) |  | 15 | (21.4) |  |  |
| Autumn | 5 | (10.0) |  | 19 | (27.1) |  |  |
| Winter | 14 | (28.0) |  | 17 | (24.3) |  |  |
| Tumor location |  |  |  |  |  | *0.247* |  |
| Upper thoracic | 2 | (4.0) |  | 9 | (12.9) |  |  |
| Middle thoracic | 26 | (52.0) |  | 31 | (44.3) |  |  |
| Lower thoracic | 22 | (44.0) |  | 30 | (42.9) |  |  |
| TNM |  |  |  |  |  | *0.855* |  |
| Stage I | 4 | (8.0) |  | 8 | (11.4) |  |  |
| Stage II | 16 | (32.0) |  | 20 | (28.6) |  |  |
| Stage III | 30 | (60.0) |  | 42 | (60.0) |  |  |
| Risk index | *1.98 (1.31, 2.29)^*^* | |  | *2.10 (1.51, 2.60)^*^* | | *0.107* |  |
| Low^#^ | 17 | (34.0) |  | 18 | (25.7) |  |  |
| High^#^ | 33 | (66.0) |  | 52 | (74.3) |  |  |

^*^ The italic numbers were the medians, 25^th^ and 75^th^ percentiles, respectively.

^#^ The median value of risk index in controls (see supplementary file 2) were used as the cut-off for low and high categories definition.

^†^ Fisher exact tests and Mann-Whitney U tests were performed for categorical and numerical data, respectively.
