# Supplementary material for: Characteristics and interplay of esophageal microbiota in esophageal squamous cell carcinoma
Source: BMC Cancer. 2022 Jun 24;22:696. doi: 10.1186/s12885-022-09771-2 (PMC9229141; doi:10.1186/s12885-022-09771-2)
Supplement: Supplementary file 6 — Additional file 6: Table S4. The corresponding description of the differential MetaCyc metabolic pathways. [file 12885_2022_9771_MOESM6_ESM.docx]

Table S4. The corresponding description of the differential MetaCyc metabolic pathways.

| MetaCyc pathway | Description |
| --- | --- |
| PWY-3661 | glycine betaine degradation I |
| PWY-7431 | aromatic biogenic amine degradation (bacteria) |
| PWY-1882 | superpathway of C1 compounds oxidation to CO2 |
| PWY-5265 | peptidoglycan biosynthesis II (staphylococci) |
| PWY-6565 | superpathway of polyamine biosynthesis III |
| PWY-6731 | starch degradation III |
| PWY-6906 | chitin derivatives degradation |
| PWY-7391 | isoprene biosynthesis II (engineered) |
