# Supplementary figures and images for: Characteristics and interplay of esophageal microbiota in esophageal squamous cell carcinoma
Source: BMC Cancer. 2022 Jun 24;22:696. doi: 10.1186/s12885-022-09771-2 (PMC9229141; doi:10.1186/s12885-022-09771-2)

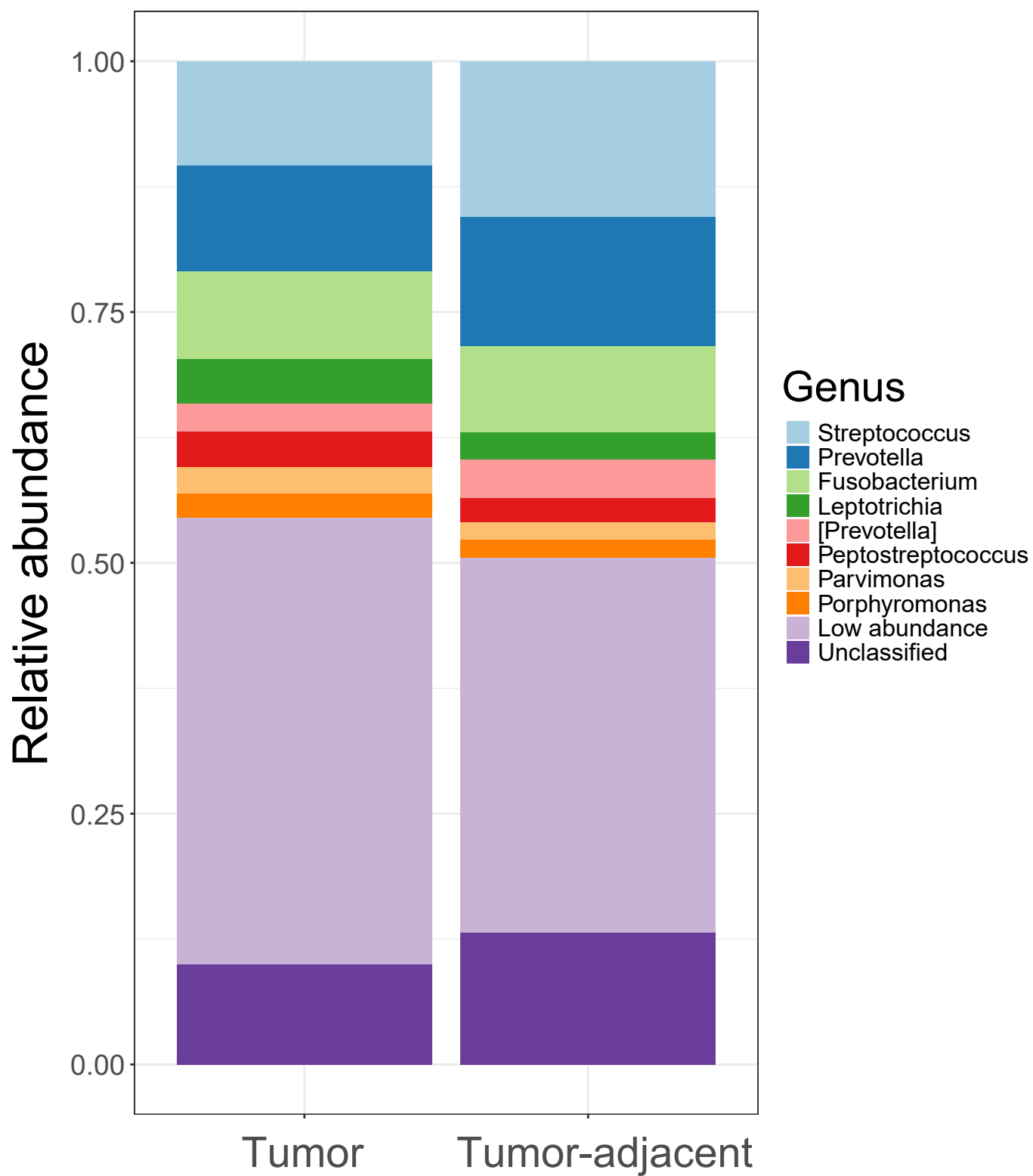

Supplement: Supplementary file 7 — Additional file 7: Fig. S1. Microbial relative abundances at the genus level in tumor and tumor-adjacent tissues. [file 12885_2022_9771_MOESM7_ESM.pdf]
